# Supplementary material for: FERN – a Java framework for stochastic simulation and evaluation of reaction networks
Source: BMC Bioinformatics. 2008 Aug 29;9:356. doi: 10.1186/1471-2105-9-356 (PMC2553347; doi:10.1186/1471-2105-9-356)
Supplement: Additional file 1 — FERN distribution, Version 1.3. This archive contains the FERN source code and binaries as well as documentation and example models in FernML and SBML. [file 1471-2105-9-356-S1.zip › fern/doc/javadoc/fern/tools/gnuplot/GnuPlot.html]

GnuPlot


---


|  |  |  |
| --- | --- | --- |
| **Overview** \| **Package** \| **Class** \| **Use** \| **Tree** \| **Deprecated** \| **Index** \| **Help** | |  |
| **PREV CLASS**   **NEXT CLASS** | **FRAMES**    **NO FRAMES**     **All Classes** |  |
| SUMMARY: NESTED \| FIELD \| CONSTR \| METHOD | DETAIL: FIELD \| CONSTR \| METHOD |  |


---


## fern.tools.gnuplot Class GnuPlot

```
java.lang.Object
  java.awt.Component
      java.awt.Container
          java.awt.Window
              java.awt.Frame
                  javax.swing.JFrame
                      fern.tools.gnuplot.GnuPlot
```

**All Implemented Interfaces:**: ImageObserver, MenuContainer, Serializable, Accessible, RootPaneContainer, WindowConstants

---

``` public class GnuPlot extends JFrame ```

The class `GnuPlot` provides methods for handling gnuplot data. Additionally
this class can be used as an interface to gnuplot in order to actually plot data.  
After data has been added by one of the `addData` methods, you are able either
to retrieve the gnuplot data file (as String - `getData` - or saved to a file -
`saveData`) or to `plot` the data by invoking gnuplot.
Once the plot is created, it can be retrieved as an Image object by `getImage`,
saved to a png file by `saveImage` or presented interactively to the screen
(by setting the visible property to true).

Multiple data can be added as well as another gnuplot object can be merged.

If the plot is shown in the `JFrame`, it can be saved by rightclicking on the frame or
pressing F3 / CTRL-S.

**Author:**
:   Florian Erhard

**See Also:**: `Axes`, Serialized Form

---

**Nested Class Summary**

**Nested classes/interfaces inherited from class javax.swing.JFrame**

`JFrame.AccessibleJFrame`

**Nested classes/interfaces inherited from class java.awt.Frame**

`Frame.AccessibleAWTFrame`

**Nested classes/interfaces inherited from class java.awt.Window**

`Window.AccessibleAWTWindow`

**Nested classes/interfaces inherited from class java.awt.Container**

`Container.AccessibleAWTContainer`

**Nested classes/interfaces inherited from class java.awt.Component**

`Component.AccessibleAWTComponent, Component.BaselineResizeBehavior, Component.BltBufferStrategy, Component.FlipBufferStrategy`


| **Field Summary** | |
| --- | --- |
| `protected  List<Axes>` | `axes` |
| `protected  List<Axes>` | `clearedAxes` |
| `protected  List<String>` | `commands` |
| `protected  String` | `defaultStyle` |
| `protected  BufferedImage` | `img` |

**Fields inherited from class javax.swing.JFrame**

`accessibleContext, EXIT_ON_CLOSE, rootPane, rootPaneCheckingEnabled`

**Fields inherited from class java.awt.Frame**

`CROSSHAIR_CURSOR, DEFAULT_CURSOR, E_RESIZE_CURSOR, HAND_CURSOR, ICONIFIED, MAXIMIZED_BOTH, MAXIMIZED_HORIZ, MAXIMIZED_VERT, MOVE_CURSOR, N_RESIZE_CURSOR, NE_RESIZE_CURSOR, NORMAL, NW_RESIZE_CURSOR, S_RESIZE_CURSOR, SE_RESIZE_CURSOR, SW_RESIZE_CURSOR, TEXT_CURSOR, W_RESIZE_CURSOR, WAIT_CURSOR`

**Fields inherited from class java.awt.Component**

`BOTTOM_ALIGNMENT, CENTER_ALIGNMENT, LEFT_ALIGNMENT, RIGHT_ALIGNMENT, TOP_ALIGNMENT`

**Fields inherited from interface javax.swing.WindowConstants**

`DISPOSE_ON_CLOSE, DO_NOTHING_ON_CLOSE, HIDE_ON_CLOSE`

**Fields inherited from interface java.awt.image.ImageObserver**

`ABORT, ALLBITS, ERROR, FRAMEBITS, HEIGHT, PROPERTIES, SOMEBITS, WIDTH`


**Constructor Summary**

`GnuPlot()`
  
          The constructor sets the DefaultCloseOperation of this JFrame to DISPOSE\_ON\_CLOSE.


| **Method Summary** | |
| --- | --- |
| `void` | `addCommand(String... command)`             Adds commands to the list of commands which are given to gnuplot by invoking `plot` |
| `Axes` | `addData(Axes axes)`             Adds data in order to plot it. |
| `Axes` | `addData(Collection col, String[] dataLabels, String[] styles)`             Adds data in order to plot it. |
| `Axes` | `addData(Object[] arr, boolean transposed, String[] dataLabels, String[] styles)`             Adds data in order to plot it. |
| `Axes` | `addData(Object arr, String[] dataLabels, String[] styles)`             Adds data in order to plot it. |
| `void` | `clearData()`             Clears the loaded data. |
| `List<Axes>` | `getAxes()`             Returns the Axes object containing the data, labels and styles. |
| `Collection<String>` | `getCommands()`             Returns the list of commands, e.g. if you want to remove some command from it. |
| `List<String>` | `getData()`             Returns the data in gnuplot data file format as list containing the data for each added axes. |
| `String` | `getDefaultStyle()`             The defaultStyle is used, when no style is defined for an column in an axes object. |
| `BufferedImage` | `getImage()`             Return the plotted data as image. |
| `String` | `getPlotCommand(List<File> files)`             Returns the plot command for a given filename |
| `boolean` | `isAccessible()`             Returns whether or not gnuplot is accessible |
| `void` | `merge(GnuPlot gnuplot)`             Merges another gnuplot object with this one by add its axes and copy its default style to the axes (if they have no style). |
| `void` | `paint(Graphics g)` |
| `void` | `plot()`             Calls gnuplot to plot the data. |
| `List<File>` | `saveData(File file)`             Saves the gnuplot data to one ore more files. |
| `void` | `saveImage(File file)`             Saves the plotted data to a png file. |
| `void` | `setDefaultStyle(String defaultStyle)`             The defaultStyle is used, when no style is defined for an column in an axes object. |

**Methods inherited from class javax.swing.JFrame**

`addImpl, createRootPane, frameInit, getAccessibleContext, getContentPane, getDefaultCloseOperation, getGlassPane, getGraphics, getJMenuBar, getLayeredPane, getRootPane, getTransferHandler, isDefaultLookAndFeelDecorated, isRootPaneCheckingEnabled, paramString, processWindowEvent, remove, repaint, setContentPane, setDefaultCloseOperation, setDefaultLookAndFeelDecorated, setGlassPane, setIconImage, setJMenuBar, setLayeredPane, setLayout, setRootPane, setRootPaneCheckingEnabled, setTransferHandler, update`

**Methods inherited from class java.awt.Frame**

`addNotify, getCursorType, getExtendedState, getFrames, getIconImage, getMaximizedBounds, getMenuBar, getState, getTitle, isResizable, isUndecorated, remove, removeNotify, setCursor, setExtendedState, setMaximizedBounds, setMenuBar, setResizable, setState, setTitle, setUndecorated`

**Methods inherited from class java.awt.Window**

`addPropertyChangeListener, addPropertyChangeListener, addWindowFocusListener, addWindowListener, addWindowStateListener, applyResourceBundle, applyResourceBundle, createBufferStrategy, createBufferStrategy, dispose, getBufferStrategy, getFocusableWindowState, getFocusCycleRootAncestor, getFocusOwner, getFocusTraversalKeys, getGraphicsConfiguration, getIconImages, getInputContext, getListeners, getLocale, getModalExclusionType, getMostRecentFocusOwner, getOwnedWindows, getOwner, getOwnerlessWindows, getToolkit, getWarningString, getWindowFocusListeners, getWindowListeners, getWindows, getWindowStateListeners, hide, isActive, isAlwaysOnTop, isAlwaysOnTopSupported, isFocusableWindow, isFocusCycleRoot, isFocused, isLocationByPlatform, isShowing, pack, postEvent, processEvent, processWindowFocusEvent, processWindowStateEvent, removeWindowFocusListener, removeWindowListener, removeWindowStateListener, reshape, setAlwaysOnTop, setBounds, setBounds, setCursor, setFocusableWindowState, setFocusCycleRoot, setIconImages, setLocationByPlatform, setLocationRelativeTo, setMinimumSize, setModalExclusionType, setSize, setSize, setVisible, show, toBack, toFront`

**Methods inherited from class java.awt.Container**

`add, add, add, add, add, addContainerListener, applyComponentOrientation, areFocusTraversalKeysSet, countComponents, deliverEvent, doLayout, findComponentAt, findComponentAt, getAlignmentX, getAlignmentY, getComponent, getComponentAt, getComponentAt, getComponentCount, getComponents, getComponentZOrder, getContainerListeners, getFocusTraversalPolicy, getInsets, getLayout, getMaximumSize, getMinimumSize, getMousePosition, getPreferredSize, insets, invalidate, isAncestorOf, isFocusCycleRoot, isFocusTraversalPolicyProvider, isFocusTraversalPolicySet, layout, list, list, locate, minimumSize, paintComponents, preferredSize, print, printComponents, processContainerEvent, remove, removeAll, removeContainerListener, setComponentZOrder, setFocusTraversalKeys, setFocusTraversalPolicy, setFocusTraversalPolicyProvider, setFont, transferFocusBackward, transferFocusDownCycle, validate, validateTree`

**Methods inherited from class java.awt.Component**

`action, add, addComponentListener, addFocusListener, addHierarchyBoundsListener, addHierarchyListener, addInputMethodListener, addKeyListener, addMouseListener, addMouseMotionListener, addMouseWheelListener, bounds, checkImage, checkImage, coalesceEvents, contains, contains, createImage, createImage, createVolatileImage, createVolatileImage, disable, disableEvents, dispatchEvent, enable, enable, enableEvents, enableInputMethods, firePropertyChange, firePropertyChange, firePropertyChange, firePropertyChange, firePropertyChange, firePropertyChange, firePropertyChange, firePropertyChange, firePropertyChange, getBackground, getBaseline, getBaselineResizeBehavior, getBounds, getBounds, getColorModel, getComponentListeners, getComponentOrientation, getCursor, getDropTarget, getFocusListeners, getFocusTraversalKeysEnabled, getFont, getFontMetrics, getForeground, getHeight, getHierarchyBoundsListeners, getHierarchyListeners, getIgnoreRepaint, getInputMethodListeners, getInputMethodRequests, getKeyListeners, getLocation, getLocation, getLocationOnScreen, getMouseListeners, getMouseMotionListeners, getMousePosition, getMouseWheelListeners, getName, getParent, getPeer, getPropertyChangeListeners, getPropertyChangeListeners, getSize, getSize, getTreeLock, getWidth, getX, getY, gotFocus, handleEvent, hasFocus, imageUpdate, inside, isBackgroundSet, isCursorSet, isDisplayable, isDoubleBuffered, isEnabled, isFocusable, isFocusOwner, isFocusTraversable, isFontSet, isForegroundSet, isLightweight, isMaximumSizeSet, isMinimumSizeSet, isOpaque, isPreferredSizeSet, isValid, isVisible, keyDown, keyUp, list, list, list, location, lostFocus, mouseDown, mouseDrag, mouseEnter, mouseExit, mouseMove, mouseUp, move, nextFocus, paintAll, prepareImage, prepareImage, printAll, processComponentEvent, processFocusEvent, processHierarchyBoundsEvent, processHierarchyEvent, processInputMethodEvent, processKeyEvent, processMouseEvent, processMouseMotionEvent, processMouseWheelEvent, removeComponentListener, removeFocusListener, removeHierarchyBoundsListener, removeHierarchyListener, removeInputMethodListener, removeKeyListener, removeMouseListener, removeMouseMotionListener, removeMouseWheelListener, removePropertyChangeListener, removePropertyChangeListener, repaint, repaint, repaint, requestFocus, requestFocus, requestFocusInWindow, requestFocusInWindow, resize, resize, setBackground, setComponentOrientation, setDropTarget, setEnabled, setFocusable, setFocusTraversalKeysEnabled, setForeground, setIgnoreRepaint, setLocale, setLocation, setLocation, setMaximumSize, setName, setPreferredSize, show, size, toString, transferFocus, transferFocusUpCycle`

**Methods inherited from class java.lang.Object**

`clone, equals, finalize, getClass, hashCode, notify, notifyAll, wait, wait, wait`

**Methods inherited from interface java.awt.MenuContainer**

`getFont, postEvent`

**Field Detail**

### img

```
protected BufferedImage img
```

---


### commands

```
protected List<String> commands
```

---


### axes

```
protected List<Axes> axes
```

---


### clearedAxes

```
protected List<Axes> clearedAxes
```

---


### defaultStyle

```
protected String defaultStyle
```


**Constructor Detail**

### GnuPlot

```
public GnuPlot()
```

:   The constructor sets the DefaultCloseOperation of this JFrame to DISPOSE\_ON\_CLOSE.
    Additionally the listeners for saving are created.


**Method Detail**

### addCommand

```
public void addCommand(String... command)
```

:   Adds commands to the list of commands which are given to gnuplot by invoking `plot`

    :   **Parameters:**: `command` - the gnuplot command (e.g. set xrange [0:100])

---


### getCommands

```
public Collection<String> getCommands()
```

:   Returns the list of commands, e.g. if you want to remove some command from it.

    :   **Returns:**: the list of gnuplot commands

---


### clearData

```
public void clearData()
```

:   Clears the loaded data.

---


### addData

```
public Axes addData(Collection col,
                    String[] dataLabels,
                    String[] styles)
```

:   Adds data in order to plot it. The data has to be given as a collection of Numbers,
    Doubles, Integer,... or as double[], int[], ... The type is inferred by using
    the java reflection api.

    :   **Parameters:**: `col` - the collections array with the data: `dataLabels` - label for each value column: `styles` - additional styles for each value column **Returns:**: axes object containing the data

---


### addData

```
public Axes addData(Axes axes)
```

:   Adds data in order to plot it. The data has to be given as an axes object.

    :   **Parameters:**: `axes` - an axes object with data to plot **Returns:**: axes object containing the data

---


### addData

```
public Axes addData(Object arr,
                    String[] dataLabels,
                    String[] styles)
```

:   Adds data in order to plot it. The data has to be given as a matrix of double, int, ...
    The first index of the matrix gives the row, the second the column. The type is inferred by using
    the java reflection api.
    In lineTypes and dataLabels you can give line style
    and labels for each column.

    :   **Parameters:**: `arr` - the data matrix to plot: `styles` - additional styles for each value column: `dataLabels` - label for each value column **Returns:**: axes object containing the data

---


### addData

```
public Axes addData(Object[] arr,
                    boolean transposed,
                    String[] dataLabels,
                    String[] styles)
```

:   Adds data in order to plot it. The data has to be given as a matrix of double, int, ...
    If transposed=true, the first index of the matrix gives the row, the second the column.
    The type is inferred by using
    the java reflection api.
    In lineTypes and dataLabels you can give line style
    and labels for each column.

    :   **Parameters:**: `arr` - the data matrix to plot: `transposed` - if the given matrix is transposed: `styles` - additional styles for each value column: `dataLabels` - label for each value column **Returns:**: axes object containing the data

---


### merge

```
public void merge(GnuPlot gnuplot)
```

:   Merges another gnuplot object with this one by add its axes and copy its
    default style to the axes (if they have no style).

    :   **Parameters:**: `gnuplot` - the gnuplot object to merge with

---


### getAxes

```
public List<Axes> getAxes()
```

:   Returns the Axes object containing the data, labels and styles.

    :   **Returns:**: Axes object

---


### getPlotCommand

```
public String getPlotCommand(List<File> files)
```

:   Returns the plot command for a given filename

    :   **Parameters:**: `files` - a list (parallel to axes) of files containing the gnuplot data **Returns:**: plot command to use in gnuplot

---


### getImage

```
public BufferedImage getImage()
```

:   Return the plotted data as image. The plot method has to be invoked first, otherwise null is returned.

    :   **Returns:**: the plotted data

---


### saveImage

```
public void saveImage(File file)
               throws IOException
```

:   Saves the plotted data to a png file. The plot method has to be invoked first.

    :   **Parameters:**: `file` - the file to save the png file **Throws:**: `IOException`

---


### getData

```
public List<String> getData()
```

:   Returns the data in gnuplot data file format as list containing the data for each added axes.

    :   **Returns:**: gnuplot data

---


### saveData

```
public List<File> saveData(File file)
                    throws IOException
```

:   Saves the gnuplot data to one ore more files. If only one data axes has been loaded,
    the file is named as given. If there are more than one, the number is attached to the
    given filename.

    :   **Parameters:**: `file` - where to save to file **Returns:**: list of the saved files **Throws:**: `IOException`

---


### isAccessible

```
public boolean isAccessible()
```

:   Returns whether or not gnuplot is accessible

    :   **Returns:**: whether or not gnuplot is accessible

---


### plot

```
public void plot()
          throws IOException
```

:   Calls gnuplot to plot the data. `loadData` has
    to be invoked first. A temporary file is created in the working directory
    to hold the data (for compatibility
    with cygwin, this file is not created by `File.createTempFile`)
    and deleted after plotting. If the JFrame is visible, it will be repainted.
    The commands are given to gnuplot via its stdin, the plotted image is retrieved
    via its stdout.

    The name(s) of the temporary files are `hashCode()i.gnuplot`. If there
    is only one file created, `i` is omitted.

    :   **Throws:**: `IOException`

---


### paint

```
public void paint(Graphics g)
```

:   **Overrides:**: `paint` in class `Container`

---


### getDefaultStyle

```
public String getDefaultStyle()
```

:   The defaultStyle is used, when no style is defined for an column in an axes object.

    :   **Returns:**: the defaultStyle

---


### setDefaultStyle

```
public void setDefaultStyle(String defaultStyle)
```

:   The defaultStyle is used, when no style is defined for an column in an axes object.

    :   **Parameters:**: `defaultStyle` - the defaultStyle to set


---


|  |  |  |
| --- | --- | --- |
| **Overview** \| **Package** \| **Class** \| **Use** \| **Tree** \| **Deprecated** \| **Index** \| **Help** | |  |
| **PREV CLASS**   **NEXT CLASS** | **FRAMES**    **NO FRAMES**     **All Classes** |  |
| SUMMARY: NESTED \| FIELD \| CONSTR \| METHOD | DETAIL: FIELD \| CONSTR \| METHOD |  |


---
